# “Short agonist stop” protocol, an ovarian stimulation for poor responders in *in vitro* fertilization (IVF): A pilot study

**DOI:** 10.3389/fendo.2022.1056520

**Published:** 2022-11-17

**Authors:** Charlotte Mauries, Noemie Ranisavljevic, Caroline Mollevi, Cecile Brunet, Samir Hamamah, Sophie Brouillet, Tal Anahory

**Affiliations:** ^1^ Department of Reproductive Medicine, Montpellier University Hospital, University of Montpellier, Montpellier, France; ^2^ Institute Desbrest of Epidemiology and Public Health, Montpellier University Hospital, University of Montpellier, INSERM, Montpellier, France; ^3^ Department of Reproductive Biology-CECOS, Montpellier University Hospital, University of Montpellier, Montpellier, France; ^4^ Embryo Development Fertility Environment, University of Montpellier, INSERM 1203, Montpellier, France

**Keywords:** ART, IVF, poor responders, short agonist stop protocol, POSEIDON criteria, ovarian stimulation protocol

## Abstract

**Introduction:**

Poor responder patients remain a challenge in assisted reproductive technologies. The “short agonist stop” (SAS) stimulation protocol uses a double stimulation (flare up effect with the gonadotropin-releasing hormone (GnRH) agonist (GnRH-a) then gonadotropins) associated with a less strenuous blockage (discontinuation of GnRH-a) to favor follicular recruitment in order to obtain a better ovarian response. This study aims to compare the number of oocytes obtained after a SAS stimulation protocol with those obtained after the previous stimulation protocol, in the same women, with poor ovarian response (POR) diagnosed according to the POSEIDON criteria.

**Design:**

This therapeutic observational retrospective cohort from 2018 to 2022, with a case-control evaluation compared with the same patients’ previous performance, included women with POR undergoing IVF with SAS stimulation protocol. The primary outcome was the number of total oocytes recovered and secondary outcomes were the numbers of mature oocytes, total embryos observed at day 2 and usable cleaved embryos and blastocysts (day 5/6).

**Results:**

63 patients with SAS and previous cycles were included. In the SAS group, the mean number of oocytes was significantly higher: 7.3 vs 5.7, p=0.018 in comparison with the previous attempt. So was the number of mature oocytes (5.8 vs 4.1, p=0.032) and the total mean number of embryos obtained at day 2 (4.1 versus 2.7, p=0.016). The SAS stimulation generated 84 usable embryos: 57 cleaved embryos and 27 blastocysts. The mean number of usable embryos was similar in both groups (1.64 vs 1.31, respectively, p=0.178). In total, out of 63 patients, after the SAS protocol, and subsequent embryo transfers (fresh and frozen, n=54), 9 patients had ongoing pregnancies and no miscarriage occurred. The cumulative ongoing pregnancy rate (cOPR) after the SAS protocol was 14.3% (9/63) per oocyte pick-up and 16.7% (9/54) per transfer.

**Conclusion:**

SAS stimulation is a short and original protocol strengthening the therapeutic arsenal of poor responders, that may offer promising results for those patients with low prognosis and previous failed IVF. Results must be confirmed with a randomized controlled trial.

## Introduction

Poor responder patients remain a challenge in assisted reproductive technologies (ART). The definition of this heterogeneous group has long been debated. In 2011, the European Society of Human Reproduction and Embryology (ESHRE) reached a consensus on the definition of ‘poor ovarian response’ (POR) to ovarian stimulation called the Bologna criteria: at least two out of three features must be present: advanced maternal age (≥40 years), a previous POR (≤3 oocytes) with a conventional stimulation protocol or an abnormal ovarian reserve test (i.e. antral follicular count (AFC) <7 follicles or anti-Müllerian hormone (AMH) <1.1 ng/ml) (1). More recently, POSEIDON’s classification (2016) enables a new definition of these low prognosis patients regarding their ability to produce at least one euploid embryo, dividing them into 4 subgroups according to qualitative and quantitative parameters: group 1: age < 35, AMH ≥ 1.2 ng/ml, AFC ≥ 5, number of oocytes retrieved ≤ 9 in the previous cycle; group 2: the same but age ≥ 35; group 3: age < 35, AMH < 1.2, AFC < 5; group 4: age ≥ 35, AMH < 1.2, AFC < 5 (2).

The POSEIDON stratification attempts to differentiate between relevant subpopulations of poor responders, for whom specific interventions might be beneficial in a more tailored and efficient care, facilitating the evaluation in clinical trials of strategies that could generate higher success in ART for those specific subgroups of patients (3).

Patients identified as “poor responders” are an increasing population representing 10 to 24% of women involved in ART (4). The optimal treatment suited for poor responders is not clearly established yet (5, 6). Gonadotropin releasing hormone (GnRH) antagonists and GnRH agonists (GnRH-a) are equally recommended for predicted poor responders (7), according to ESHRE recommendations (Ovarian stimulation for IVF/ICSI, Guideline of the ESHRE, 2019). Also, consideration should be given to a mild ovarian-stimulation protocol (8) and dual stimulation protocol in the same ovarian cycle, both offering encouraging results for POR (9). The efficiency of adjuvant co treatments has not yet been proven, although dehydroepiandrosterone (DHEA), LH or coenzyme Q10 supplementation seems promising (10, 11). New hopes arise from groundbreaking treatments in development such as autologous platelet-rich plasma intra ovarian injection (12) or *in vitro* activation of follicles (13). Finally, when ART is unsuccessful with autologous oocytes, egg donation, associated with a high live birth rate (LBR), remains the best option for poor responders.

Their care in ART remains a challenge and the efficiency of their stimulation protocol is still being discussed.

**Figure 1:**
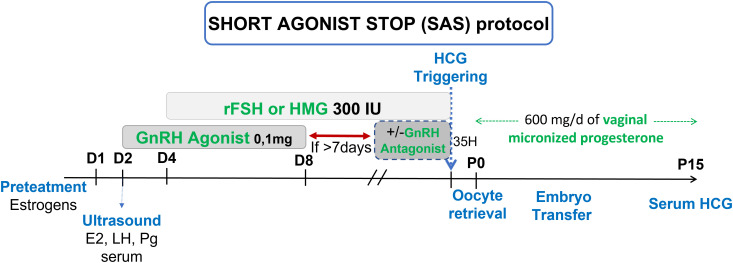
Short agonist stop (SAS) stimulation protocol.

Schachter et al. and Hazout et al. hypothesized that POR benefits from a double stimulation (flare up effect then gonadotropins) associated with a less strenuous blockage (discontinuation of GnRH-a) to favor follicular recruitment in order to obtain a better ovarian response and produce more oocytes and embryos, including more usable embryos, increasing chances of ongoing pregnancies (OP) in these low prognosis patients (14, 15). Based on this data, we proposed to our poor responders patients the “Short agonist stop” (SAS) protocol that uses GnRH-a at first for the flare up effect at the beginning of the cycle for 7 days in total then stopped, enabling pituitary desensitization in order to prevent a premature LH surge, associated with a controlled ovarian stimulation with gonadotropins at maximum dosage (300IU/d) (15–18) (**Figure 1**).

The SAS protocol as presented in this study is not mentioned in recent literature about stimulation protocols and management of poor responders in ART (19, 20), nor in ESHRE’s guidelines about ovarian stimulation for IVF/ICSI (21). This type of stimulation was studied more often in the 2000s, but still, only one randomized controlled trial (RCT) used this protocol (15).

Therefore, this pilot study was performed to evaluate if in poor responders’ patients, the SAS stimulation protocol allows for a better number of oocytes (primary outcome), mature oocytes, total embryos at D2 and usable embryos (secondary outcomes) in comparison with the last previous IVF attempt within the same patients, in order to obtain more ongoing pregnancies in those patients with numerous previous failed IVF attempts.

## Materials and methods

### Study design

This was an observational, retrospective, single-center study with case-control evaluation compared with the same patients’ previous performance in their last IVF attempt. All patients enrolled in an IVF protocol with the “short agonist stop” (SAS) stimulation protocol at Montpellier University Hospital between January 1^st^, 2018, and January 1^st^, 2022 (with follow-up until April 1^st^, 2022) were included.

The main objective of the study was to compare, in poor responders, the number of oocytes obtained after the SAS stimulation protocol versus previous classical stimulation of their last IVF attempt, each patient being their own control.

### Study outcomes

The main outcome measure was the total number of oocytes obtained in poor responder patients after the SAS stimulation protocol.

Secondary outcome measures were the total number of mature oocytes, embryos at day 2, usable embryos (cleaved embryos or blastocysts, eligible for transfer: either fresh or frozen, or surplus embryos, still frozen), cancellation and freeze all rates, and the outcome of IVF attempt: no pregnancy, biochemical pregnancy, miscarriage, ectopic pregnancy, ongoing pregnancy (rate per cycle, per oocyte pick up and per transfer) and live birth (if available), including cumulative outcomes.

### Patients

Patients included in the study were ≥ 18 and < 43 years old. Preimplantation genetic testing and fertility preservation cycles were excluded. Non-compliance to the SAS protocol (modification of duration of GnRH-a) and lacking data in previous cycle were exclusion criteria. Only the first cycle with SAS was included compared with the previous stimulation cycle. Women with defined POR (low prognosis patients) were included, according to POSEIDON stratification.

Registered patient data included age, AMH rate, AFC, body mass index (BMI), POSEIDON’s group and the rank of the attempt. Measured parameters included type of protocol, days of stimulation, dose and type of gonadotropin, total dose used of gonadotropin, and all outcome measures as detailed above.

### Ethical approval

Patients were informed of the investigation and gave their consent before participation. This study was approved by the local Ethics Committee (IRB registration number 202201068).

### Previous IVF protocol (first protocol group)

**Figure 2:**
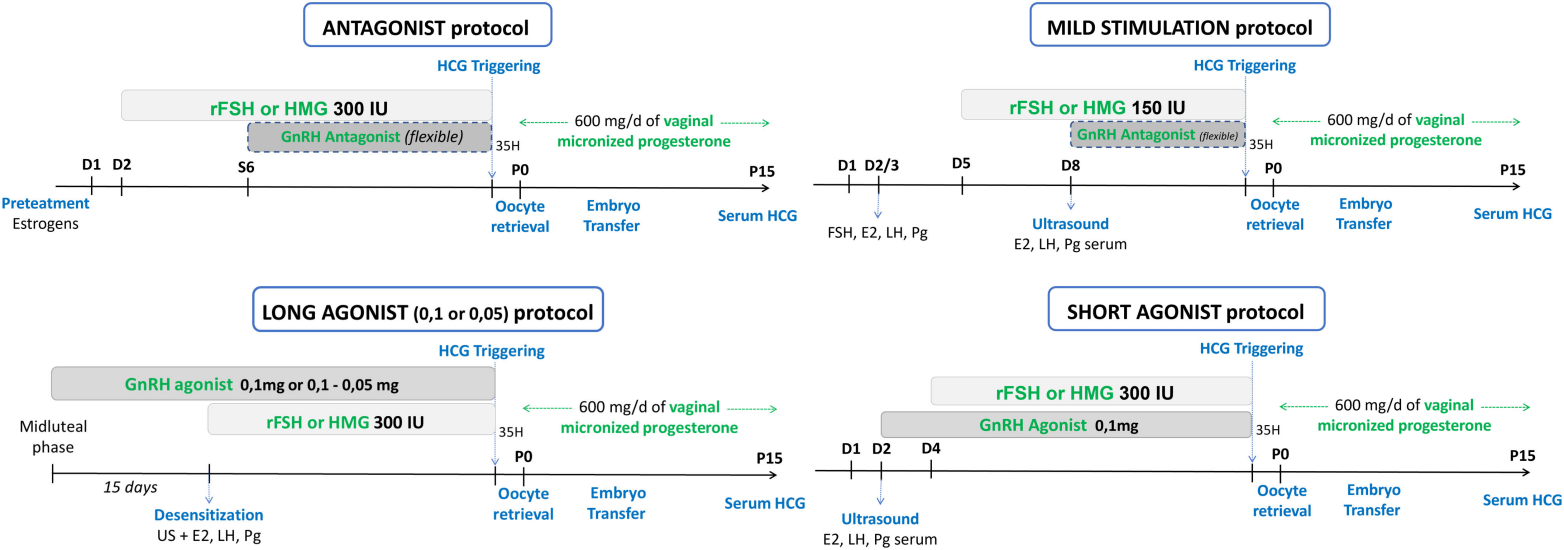
Previous standard protocol for POR: antagonist protocol, long and short agonist protocol, mild stimulation.

Enrolled patients were treated in two consecutive cycles. The first attempt was achieved with a standard protocol for POR: antagonist protocol, long or short agonist protocol and mild stimulation, summarized in **Figure 2**.

Ovulation triggering was performed with recombinant hCG (Ovitrelle^®^, 250 mg, Laboratoire Merck, France) when the leading follicles (at least 3 follicles, except for mild stimulation) reached a mean diameter of 17 mm and oocyte retrieval was performed 35 hours after. If less than 3 follicles were recruited, a conversion to ovulation induction or intra uterine insemination was performed, if possible (at least one patent tube and adequate semen parameters). Luteal phase was supported similarly by daily vaginal administration of 600 mg of micronized progesterone (Utrogestan^®^, Laboratoire Besins International, France), until the pregnancy test.

Patients for whom the standard protocol has failed (no ongoing pregnancy, no remaining cryopreserved embryo) were treated in the subsequent cycle with the SAS protocol. Clinical and laboratory aspects of treatment were mainly done in a similar fashion in both cycles if the first cycle was done in Montpellier University Hospital, each patient acting as her own control.

### Short agonist stop protocol (SAS group)

For the SAS protocol (**Figure 1**), pre-treatment with estrogens (Provames^®^, Laboratoire Sanofi-Aventis, France), starting in the midluteal phase (D20) of the preceding cycle, was prescribed. After an ultrasound to confirm ovarian quiescence and a thin endometrium, associated with low P serum on day 2 of the cycle, 0.1 mg triptorelin (Decapeptyl^®^, Laboratoire Ipsen Pharma, France) was initiated daily, for 7 days, then stopped. Controlled ovarian stimulation (COS) was initiated 2 days after the beginning of the GnRH-a, on day 4, with FSH or hMG at 300 IU as a starting dose. After 7 days without agonist, GnRH antagonist, ganirelix 0.25 mg (Orgalutran^®^, Laboratoire Organon, France) was used daily until triggering. Triggering, oocyte retrieval and luteal phase support were all performed in a similar fashion to the previous IVF attempt.

### IVF and embryo quality assessment

Conventional IVF (cIVF) or intra cytoplasmic sperm injection (ICSI) technique were used as appropriate. IVF procedure was performed in our unit as previously described (22). At D3, embryo morphology was graded using a standard system including number, size and uniformity of blastomeres, degree of fragmentation and the presence of multinucleated blastomeres. Usable cleaved embryos were defined as embryos with at least 3 blastomeres at D2 and 7 at D3, blastomeres with relative uniformity and no multinucleation, with <30% of fragmentation. At D5/6, blastocyst morphology was evaluated according to the Gardner and Schoolcraft grading system (23). Thus, usable blastocysts were defined as full (grade 3), expanded (grade 4), partially hatched (grade 5), or fully hatched (grade 6) blastocysts with at least grade B trophectoderm quality. Usable blastocysts were freshly transferred at D5 or cryopreserved at D5/6 for subsequent transfers.

### Embryo transfer and pregnancy outcome

Fresh embryo transfers were performed either at the cleavage (D2-D3) or blastocyst stage (D5). Early blastocysts (grade 1 or 2) at D5 were kept in culture until D6 and cryopreserved if considered usable at that point. The surplus embryos (D2-D3 or D5-D6) that were considered usable according to morphologic criteria were cryopreserved for subsequent transfers. The embryo transfer strategy was determined by a multidisciplinary team. Embryos were cryopreserved by vitrification and thawed following the manufacturer’s recommendations (Vit Kit-Freeze and Vit Kit-Thaw, FUJIFILM Irvine Scientific—BioCare Europe™). A maximum of two embryos were replaced.

All usable embryos were frozen (freeze all strategy) for subsequent frozen ET cycles if the circumstances were unsuitable for fresh ET, for instance in case of elevated P level, inadequate uterine cavity, prolonged ovarian stimulation (> 13 days) or accumulation of vitrified embryos for later transfer (desynchronization). FET (frozen embryo transfer) cycles were performed with natural cycle, hormonal replacement therapy or stimulated cycle regarding the ovulatory status.

Pregnancy was assessed by serum hCG assay after 15 days from oocyte retrieval. A biochemical pregnancy is characterized by the absence of an identifiable pregnancy on ultrasound examination despite a positive blood hCG pregnancy test (<100 IU/L). Clinical pregnancy was confirmed if a fetal heartbeat could be observed by transvaginal ultrasound. An ongoing pregnancy (OP) is defined as a pregnancy with a detectable heartbeat at 12 weeks of gestation or beyond. Live birth is defined as the birth of at least one living child, irrespective of the duration of gestation. Cumulative OP rate (cOPR) includes the outcomes from all fresh and frozen embryo transfers following an episode of ovarian stimulation.

### Statistical analysis

Quantitative variables were reported with the usual statistics: mean and standard deviation, median and range, and were compared between the previous IVF simulation and SAS protocols using sign rank test (paired tests were performed because each subject is their own control). Categorical variables were reported with the number of observations (N) and the frequency (%) of each modality and compared using a McNemar test (paired tests were performed because each subject is their own control). All tests were two-sided and p-values < 0.05 were considered as statistically significant. Statistical analyses were performed with STATA 15.0 (StatCorp, College Station, TX).

## Results

### Patient characteristics

**Figure 3:**
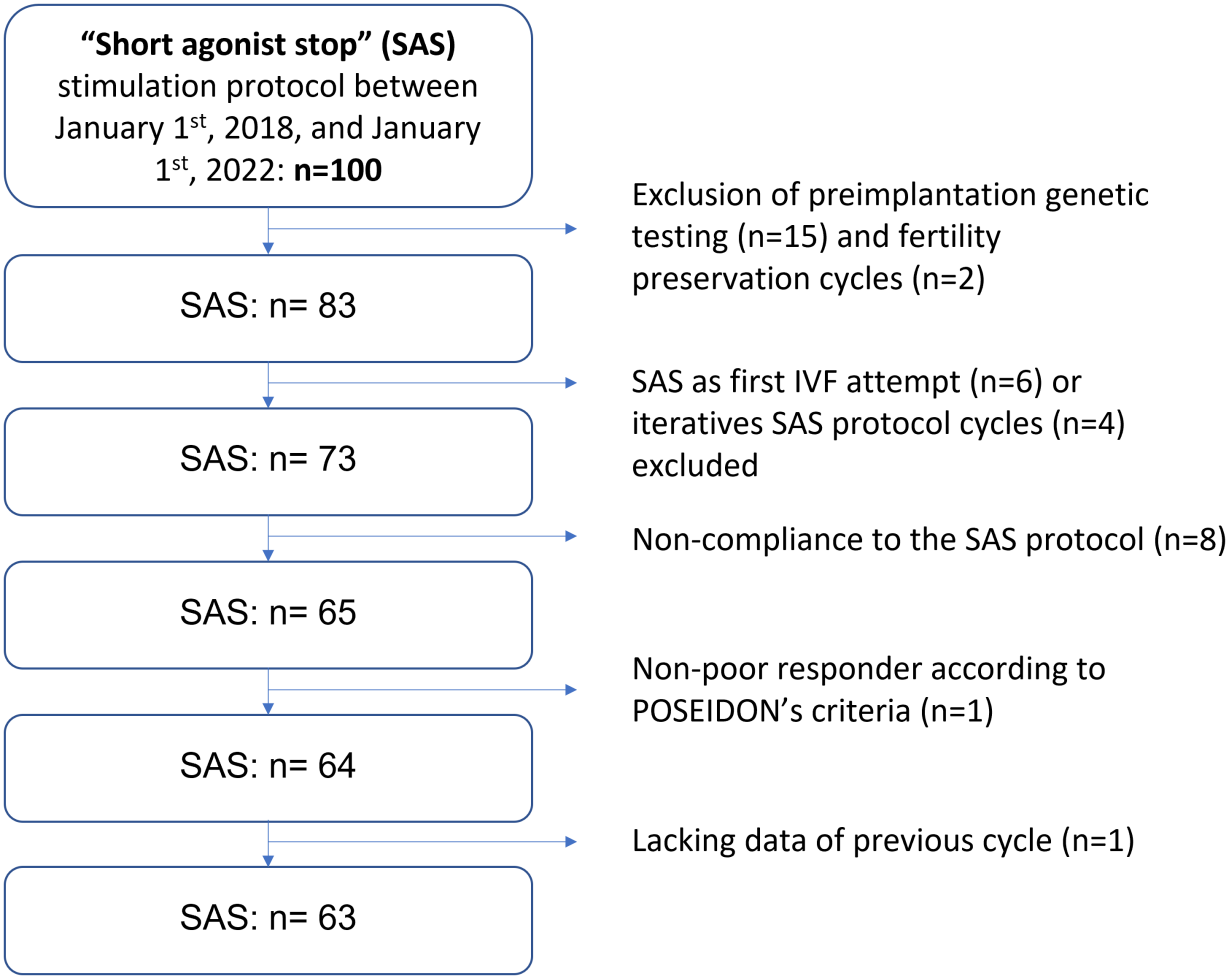
Study flow chart.

Out of 100 patients, a total of 63 patients who underwent IVF with the SAS protocol consecutively to a previous stimulation cycle were included in the final analysis (**Figure 3**). Patient characteristics are summarized in **Table 1** and treatment data in **Table 2**.

**Table 1:** Demographic and baseline characteristics at the beginning of SAS protocol.

	Overall population n = 63
**Age**	
* Mean (SD)*	36.62 (4.09)
* Median (min; max)*	37 (26; 42)
**BMI**	
* Mean (SD)*	24.26 (4.74)
* Median (min; max)*	22.41 (17.21; 35.64)
**AMH**	n=63
* Mean (SD)*	1.02 (0.43)
* Median (min; max)*	0.93 (0.03; 2.34)
**Antral follicle count (AFC)**	
* Mean (SD)*	9.86 (3.35)
* Median (min; max)*	10 (2; 16)
**POSEIDON’s group**	n (%)
* 1*	8 (12.70)
* 2*	13 (20.63)
* 3*	12 (19.05)
* 4*	30 (47.62)
**Total IVF cycles (including SAS)**	
* Mean (SD)*	2.92 (0.94)
* Median (min; max)*	3 (1; 5)
**Time between previous attempt and SAS (months)**	
* Mean (SD)*	8.14 (4.95)
* Median (min; max)*	7.36 (2.17; 28.6)

**Table 2:** Controlled ovarian stimulation and IVF cycle description.

	Previous IVF protocol n = 63	SAS protocol n = 63	*P *value
	n (%)	n (%)	
**Stimulation protocol**			**-**
* Antagonist*	48 (76.20)		
* Long Agonist 0.05*	3 (4.76)		
* Long Agonist 0.1*	8 (12.70)		
* Short Agonist*	2 (3.17)		
* Mild stimulation*	2 (3.17)		
* Short Agonist Stop*		63 (100)	
* DHEA Supplementation*	0	2 (3.17)	
**Gonadotropin**			**-**
* HMG*	42 (66.67)	50 (79.37)	
* R FSH*	21 (33.33)	13 (20.63)	
**Dose by day**			**-**
* Mean (SD)*	309.52 (51.28)	302.18 (23.28)	
* Median (min; max)*	300 (150; 450)	300 (225; 450)	
**Cancellation rate**	12 (19.05)	12 (19.05)	1.000
**Cause of cancellation**			**-**
* Insufficient ovarian response*	8 (66.67)	9 (75.00)	
* Inappropriate cycle*	2 (16.67)	1 (8.33)	
* Premature ovulation*	2 (16.67)		
* OI or IUI conversion*		2 (16.67)	
***Cycles with oocyte retrieval***	**n = 51**	**n = 51**	
**Cycle duration (days)**			
* Mean (SD)*	–	12.3 (2.03)	
* Median (min; max)*	–	12 (7; 16)	
**Total Gonadotropin dose**			
* Mean (SD)*	–	3732 (665.2)	
* Median (min; max)*	–	3600 (2087; 5250)	
**IVF or ICSI distribution**			0.092
* IVF*	14 (27.45)	6 (11.76)	
* ICSI*	37 (72.55)	45 (88.24)	
**Number of oocytes**			0.018
* Mean (SD)*	5.74 (2.92)	7.31 (3.61)	
* Median (min; max)*	5 (1; 16)	7 (2; 22)	
**Number of metaphase II oocytes**			0.032
* Mean (SD)*	4.12 (2.22)	5.80 (3.42)	
* Median (min; max)*	4 (0; 12)	5 (0; 20)	
**Number of embryos at D2**			0.016
* Mean (SD)*	2.74 (2.10)	4.06 (2.80)	
* Median (min; max)*	2 (0; 9)	4 (0; 17)	
**Freeze all rate**	7 (13.73)	24 (47.06)	<0.001
**Cause of freeze all**			**-**
* Desynchronization*	3 (42.86)	2 (8.33)	
* Elevated P serum*	1 (14.29)	2 (8.33)	
* Excessive length of stimulation*	3 (42.86)	17 (70.83)	
* HOSS risk*		1 (4.17)	
* COVID 19*		2 (8.33)	
**No usable embryo**	12 (23.53)	9 (17.65)	0.206

OI, ovulation induction; IUI, intra-uterine insemination.

Desynchronization: choice of frozen embryo transfer strategy (endometrial receptivity tests, prolonged desensitization before ET…).

Almost half of the patients belong to POSEIDON’s group 4 (47.6%). The previous stimulation protocol occurred almost 8 months (8.14, SD 4.95) before SAS and was in most cases an antagonist protocol (76.2%). HMG were mostly used compared to rFSH in both protocols: 66.7% (previous) and 79.4% (SAS).

### SAS IVF cycle description and outcomes

The cancellation rate (19.1%) was the same in both groups. It was generally due to insufficient ovarian response (less than 3 follicles recruited) (66.7% and 75.0% in the previous and SAS cycle respectively). In the SAS group, there was no cancellation due to premature ovulation. In both groups, 51 patients proceeded to oocyte retrieval (4 patients had their cycle cancelled during the first attempt and with the SAS protocol; the other 8 were different patients in each group). In both groups, ICSI was mostly performed (72.55 and 88.24%) (**Table 2**).

In the SAS group, 7.3 oocytes were retrieved versus 5.7 in the previous attempt, which was significantly higher (p=0.018). The mean number of metaphase II oocytes was also significantly higher: 5.8 versus 4.1(p=0.032) as well as the total mean number of embryos at day 2: 4.1 versus 2.7 (p=0.016). The freeze all rate was significantly higher in the SAS group: 47.1% (24/51) versus 13.7% (p=<0.001), mostly due to excessive length of stimulation (70.8%). There was no difference between groups in cycles without usable embryos (12 vs 9, p=0.206) (**Table 2**).

There was no statistically significant difference in the embryo stage for those transferred (p=0.507). The number of cumulative ET in the SAS group was higher: 54 vs 42, but with no statistical difference (p=0.124). In the previous cycle group, there were more fresh ET (40.7 vs 78.6%) than in the SAS group, which had more frozen ET (37.0 vs 14.3%). Both groups had similar ET with cleavage embryos (68.5 vs 73.8%) but in the SAS group, there were more ET with blastocysts: 24.1 vs 16.7%. The mean number of usable embryos was higher in the SAS group than in the previous IVF: 1.6 vs 1.3, but with no statistical significance (p=0.178) (**Table 3**).

**Table 3:** Cumulative IVF outcomes.

	Previous IVF protocol	SAS protocol	*P*value
Total number of transfers (fresh + frozen)	n = 66	n = 75	
	n (%)	n (%)	
**Number of cumulative ET**			0.124
* Total*	42	54	
* Mean (SD)*	0.67 (0.57)	0.86 (0.73)	
* Median (min; max)*	1 (0; 2)	1 (0; 3)	
**Type of ET**			–
* Fresh ET*	33 (78.57)	22 (40.74)	
* Frozen ET*	6 (14.29)	20 (37.04)	
* Subsequent frozen ET*	3 (7.14)	12 (22.22)	
**Embryo stage**			0.507
* Cleavage stage (D2, D3)*	31 (73.81)	37 (68.52)	
* Morula stage (D4)*		1 (1.85)	
* Blastocyst stage (D5, D6)*	7 (16.67)	13 (24.07)	
* Cleavage + Blastocyst stage (double ET)*	4 (9.52)	3 (5.56)	
**Number of cumulative usable embryos**			0.178
* Total*	67	84	
* Embryos transferred*	67	72	
* Remaining cryopreserved embryos*	0	12	
* Mean (SD)*	1.31 (0.97)	1.64 (1.55)	
* Median (min; max)*	1 (0; 4)	2 (0; 10)	
**Number of cumulative usable blastocysts**			0.266
* Total*	10	27	
* Mean (SD)*	0.20 (± 0.63)	0.53 (± 1.51)	
* Median (min; max)*	0 (0; 4)	0 (0; 10)	
**Cumulative outcomes**			
* Cancellation before oocyte pick up*	12 (18.18)	12 (16.00)	
* No usable embryo*	12 (18.18)	9 (12.00)	
* No pregnancy*	31 (46.97)	42 (56.00)	
* Biochemical pregnancy*	5 (7.58)	2 (2.67)	
* Miscarriage*	6 (9.09)	0 (0)	
* Ectopic pregnancy*	0 (0)	1 (1.33)	
* Ongoing pregnancy*	0 (0)	9 (12.00)	

ET, Embryo transfer.

In the previous attempt group, 5 biochemical pregnancies and 6 miscarriages occurred. There was no ongoing pregnancy.

No miscarriage occurred after the SAS protocol. The cOPR in the SAS group was 12.0% per cycle, 14.3% per ovarian puncture and 16.7% per transfer (**Table 4**). Out of 63 patients, after the SAS protocol and subsequent ET, 9 patients had OP and 8 of them had deliveries with live births. The remaining one is still pregnant (3^rd^ trimester). OP occurred in patients belonging to POSEIDON’s group 3 (n=5), group 4 (n=2) and group 1 (n=2).

**Table 4:** SAS cumulative IVF outcomes.

	Per cycle n = 75	Per OPU n = 63	Per transfer n = 54
	n (%)	n (%)	n (%)
**Miscarriage rate**	0 (0)	0 (0)	0 (0)
**Ongoing pregnancy rate**	9 (12.0)	9 (14.3)	9 (16.7)
**Live birth rate**	8* (10.7)	8* (12.7)	8* (14.8)

OPU, Oocyte pick-up.

*1 patient is still pregnant.

## Discussion

This retrospective study has documented that SAS stimulation is a short and original protocol strengthening the therapeutic arsenal of poor responders, which may offer promising results for those patients with low prognosis and a record of failed IVF. This protocol resulted in a significantly higher number of oocytes, mature oocytes, and embryos obtained and a non-significantly higher number of usable embryos, in comparison with their previous IVF cycle.

In Loutradis and Badawy et al.’s review, for poor responders, GnRH agonist flare up and long agonist protocols did not seem to be as advantageous as a reduction of GnRH-a doses, “stop” protocols, or microdose GnRH-a flare regimens. These regimens all appeared to improve outcomes, although the benefit of one approach over another has not been convincingly established (24, 25), with no difference between their outcomes (26). The SAS protocol is a mix of flare and “stop” protocols. Yet, a most recent RCT found that the microdose flare-up seemed to be superior to the flare-up protocol, with significantly higher LBR (p=0.036) (27), but with similar efficacy when compared to GnRH antagonist protocol (28). The advantage of SAS over long protocol is the shorter duration of stimulation which could favor better compliance and tolerance.

The use of a GnRH-a during COS in long protocols may lead to poor ovarian response due to intense endogenous FSH suppression (29) and the possible local inhibitory effect of GnRH-a on the ovaries (30). Like the microdose GnRH-a flare protocol (31), the SAS protocol may overcome these adverse effects by enhancing the release of early follicular phase FSH with the flare up effect, intensifying the effects of the exogenous gonadotrophins. Short use of GnRH-a (7 days) does not profoundly inhibit ovarian response through the ovarian GnRH receptors while sufficiently inhibiting premature LH surges (32). In the SAS group, no cancellation were observed due to premature LH surge or ovulation in the following 7 days after discontinuation of GnRH-a, as in Hazout’s RCT (15), showing the efficiency of latent agonist blockage, as shown in Pantos et al.’s study, with up to 12 days without GnRH-a (33). After stopping GnRH-a (5-day course), endogenous GnRH activity appeared to be suppressed for at least 7 days afterwards (34), because the pituitary is in a refractory state of LH secretion, as found in Cedrin-Durnerin et al.’s study, showing decreased LH concentrations after an early discontinuation of GnRH-a administration compared with a long agonist protocol (17). Indeed, hypophyseal desensitization is related to GnRH receptor reduction, leading to a progressive reduction in gonadotropin synthesis, that remains for some days (35).

In the SAS group, significantly higher oocytes and mature oocytes were retrieved than in the previous attempt. Cumulative LBR is considered to be the most important outcome in IVF and it is in direct association to oocyte yield following COS (36). In Sunkara’s study, the gap between having 1 vs 2 oocytes retrieved, or 2 vs 3, had a major impact on live birth rates: from 5 to 13% or 13 to 18% respectively in 35-37 years old patients (36). Consequently, maximizing the oocyte yield is pivotal for stimulation, so SAS protocol enabling more oocytes is paramount.

On the one hand, in a small cohort (37), poor responders undergoing ultrashort flare up GnRH-a versus GnRH-antagonist protocol also demonstrated a significantly higher number of oocytes retrieved and embryos transferred as compared with the patients’ previous IVF attempts. When discontinued GnRH-a protocol is compared with long agonist protocol in POR patients, Garcia- Velasco’s RCT found the retrieval of a significantly higher number of oocytes (38) whereas Pantos et al. found no difference in recovered oocytes (33). On the other hand, Hazout et al. utilized in their RCT a 7-day GnRH-a protocol (as the SAS) that yielded less retrieved oocytes (7.3 vs 10.7), but more embryos per cycle with markedly decreased hMG requirements when compared with long GnRH-a protocol. Yet, the number of obtained fresh embryos available for transfer and the OPRs were similar in the two treatment groups, suggesting greater efficiency of the 7-day protocol despite the smaller number of oocytes (15).

We found that the mean number of usable embryos were higher in the SAS group with no statistical significance. The number of cumulative ET in the SAS group was higher: 54 vs 42, but with no statistical difference (p=0.124). Twelve surplus embryos are waiting for ET (mainly because of an ongoing pregnancy), so the number of cumulative ET would probably be significant if all embryos were transferred, with potentially more pregnancies. Schachter et al. also found significantly more cleaving embryos with improved morphology after discontinued GnRH-a protocol in comparison with long agonist protocol (14).

The freeze all rate was significantly higher in the SAS group mostly due to prolonged stimulation, indication based on the fact that prolonged stimulation is associated with decreased ART success because of impaired endometrium for implantation (except for PCOS) (39–41). However, a recent study showed that it is in fact the total dose of gonadotropin received that impacts LBR in fresh cycles (42). So, a freeze all strategy regarding only the total gonadotropin dose received (>5000 IU) would be more appropriate. Six usable cryopreserved embryos because of prolonged stimulation were lost because of lysis (data not shown). The number of usable embryos would have been higher in the SAS group if they were freshly transferred.

The cOPR in the SAS group was 14.3% per ovarian puncture and 16.7% per transfer. One study suggests that the LBR in POR patients ranges from less than 1 to 10% per cycle (5). Other data found a cumulative LBR per ovarian puncture lower in POSEIDON patients with Group 3: 29.4% and Group 4: 12.5% (43). When discontinued GnRH-a protocol is compared with long agonist protocol in POR patients, the pregnancy rates were similar in Garcia-Velasco’s RCT (38), OPR per transfer were 24% in Faber’s study (44), and the PR in the discontinuation group of Pantos’ article was almost twice that of the long protocol (35.2% vs 19.4%) (33). Those overall results are higher than ours, but our population had numerous previous failures and a majority of patients belonging to group 4 (47.6%), with the lowest prognosis.

Otherwise, the miscarriage rate (MR) in the SAS group was particularly low, probably because of the small size of our population and of the selection of our population with previous failed IVF cycle (no pregnancy, biochemical pregnancies or miscarriages). There is no reason to believe that the SAS stimulation could reduce miscarriage by enhancing the ploidy rate, because recent studies show that ovarian stimulation does not impact the risk of aneuploidy (45, 46). Other factors like individualized luteal support with adequate progesterone levels in FET might play a role (47), as it was most recently changed in our center.

Some limits to our study must be pointed out. The main limitation is its retrospective nature and the modest sample size. Due to the design of the study, the outcome of IVF (OPR or LBR) cannot be chosen as the main outcome measure because we proceeded to another IVF attempt precisely because the previous IVF failed. The heterogeneity of the group of previous attempts is another limit. Different protocols were used with different pretreatments: estrogens priming (76.2%), daily agonist desensitization (17.46%) or no pretreatment (6.34%). IVF was sometimes performed in another center (n=16) with lacking data (like duration of the stimulation) and with different laboratory techniques, but this rather reflects the reality of poor responders’ care and patients remain the same. In addition, the cancellation rate affecting different patients (n=8) in both groups is a limit, as our main outcome was the number of oocytes retrieved, occurring after oocyte pick-up. Furthermore, the comparability of both groups is limited: the time between both attempts could be associated with some changes in variables such as BMI, obviously age (but in favor of previous attempt), AMH, sperm etc. Yet, the delay in between is sufficiently short (8.14 months, SD 4.95) that no major differences are to be expected, as found in Romanski et al.’s study, showing that the delay in IVF treatment up to 180 days does not affect pregnancy outcomes in women with diminished ovarian reserve (48). There were only 3 outliers over 18 months, and one of them achieved a live birth after SAS protocol. Furthermore, the COVID-19 crisis was partly responsible of this delay. Also, the treatment strategy is adapted regarding the fact that the previous attempt failed, in order to improve it, for example, by performing ICSI instead of conventional IVF with the aim to improve the fertilization rate, or DHEA supplementation (only 2 patients). We must point out that cycle-to-cycle variation in ovarian response exists and that the growth and steroidogenic characteristics of antral cohorts in response to exogenous FSH may vary from one cycle to another (e.g. expression and sensitivity of FSH receptors of granulosa cells (49)). However, the cycle-to-cycle heterogeneity would probably be similar in both groups.

The strength of this study lies in the comparison of the same patients’ performance in consecutive cycles, which presents the advantage of controlling for differences that might be expressed in randomized patient groups. Patients acting as their own controls (same type of infertility, responsiveness to gonadotropin, genetic predispositions and so on) make the study less biased. The stimulation protocol is original and has only been used in one published study (15). The benefice of SAS in comparison with other stimulation protocols, is that it is a more friendly protocol with a shorter duration of agonist, meaning on the one hand no time needed for desensitization and on the other hand, more time with only one injection of gonadotropin.

The results of this pilot study need to be confirmed with a prospective trial to assess the genuine usefulness of this protocol and to decipher which type of poor responders would benefit the most from SAS stimulation.

In conclusion, the SAS stimulation protocol may offer promising results (more mature oocytes and embryos) for poor responders with low prognosis and previous failed IVF. Those results must be confirmed with a large prospective study such as a RCT evaluating live birth rate after SAS protocol versus standard protocol. The SAS original protocol might strengthen the therapeutic arsenal of poor responders and enable a more tailored management.

## Data availability statement

The original contributions presented in the study are included in the article. Further inquiries can be directed to the corresponding author.

## Author contributions

CM is the main author. CM collected all the data, wrote the manuscript and designed the figures, working under the direction of TA. The methodology was elaborated in association with TA and CMo. CMo performed the statistical analysis and made the tables. NR, CB, SH, and SB were proofreaders and enhanced the manuscript with their corrections. All authors contributed to the article and approved the submitted version.

## Conflict of interest

The authors declare that the research was conducted in the absence of any commercial or financial relationships that could be construed as a potential conflict of interest.

## Publisher’s note

All claims expressed in this article are solely those of the authors and do not necessarily represent those of their affiliated organizations, or those of the publisher, the editors and the reviewers. Any product that may be evaluated in this article, or claim that may be made by its manufacturer, is not guaranteed or endorsed by the publisher.
